# Beyond Telomerase: Telomere Instability as a Novel Target for Cancer Therapy

**DOI:** 10.4172/1747-0862.1000091

**Published:** 2013-12-09

**Authors:** Maria Fadri-Moskwik, Qing Zhou, Weihang Chai

**Affiliations:** Section of Medical Sciences and School of Molecular Biosciences, Washington State University, USA

## Abstract

Telomeres are areas of heterochromatin composed of TTAGGG repeats located at the ends of linear chromosomes. They play a critical role in keeping genome stable and preventing premature aging diseases and the development of cancer. Characterizing mechanisms of telomere maintenance and understanding how their deregulation contributes to human diseases are therefore important for developing novel therapies. A key mechanism driving telomere maintenance and replicative immortality in cancer cells is telomere elongation by telomerase, and many emerging potential telomere-based therapies have focused on targeting telomerase components. By contrast, recent studies on telomere maintenance mechanism suggest that disrupting telomere stability by interfering with alternative mechanisms of telomere synthesis or protection may also yield new strategies for the treatment of cancer. This review will focus on emerging regulators of telomere synthesis or maintenance, such as G4 telomeric DNA, the CST complex, the t-loop, and shelterins, and discuss their potential as targets for anti-cancer chemotherapeutic intervention in the future.

## Telomeres are Important for Genomic Stability and Prevention of Human Diseases

In humans, telomeres are areas of heterochromatin composed of TTAGGG repeats located at the ends of linear chromosomes. Components of telomeres are TTAGGG repeats [1], nucleosomes [2], t-loop [3], and telomere binding proteins [4]. Telomeres have three functions: first, telomeres protect the ends of chromosomes and facilitate their replication by telomerase; second, telomeres prevent recognition of chromosome ends as breaks and suppress DNA damage response (DDR) [5,6]; and last, recent work suggests that telomeres are emerging as potential sensors of genotoxic stress [7]. Deregulation of telomere maintenance, or telomere instability, is directly associated with many diseases such as cancer [8], dyskeratosis congenita [9,10], idiopathic pulmonary fibrosis [11,12], Coats Plus disease [13], aplastic anemia [14], as well as bone marrow failure [15] and premature aging syndromes [16]. On a molecular level, telomere instability can lead to genomic instability and is associated with genomic defects such as telomere shortening, telomere fusions, and chromosomal rearrangements [17,18]. Therefore, it is important to understand the mechanisms of telomere maintenance and how telomere instability, which contributes to human diseases and genomic instability, may arise.

**Figure 1:**
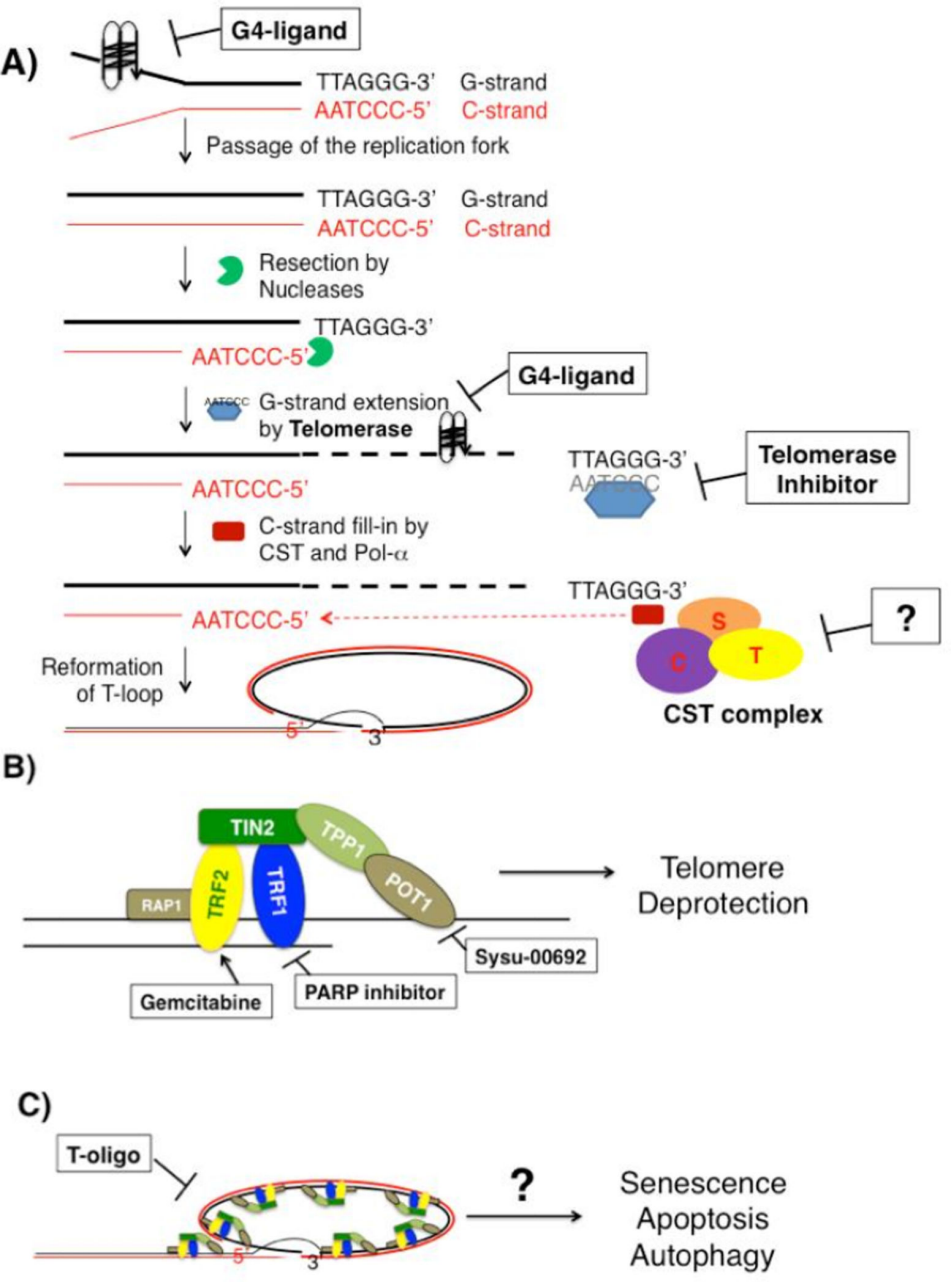
Mechanisms of Telomere Instability (A) Molecular targets that directly affect telomere integrity. G4-ligands can bind to telomere DNA and induce formation of or stabilize the G-quadruplex structure, which can block telomere DNA synthesis as well as inhibit telomerase extension of telomeres. Telomerase inhibitors that target either the hTR or hTERT subunit directly inhibit telomere extension, therefore disrupting telomere maintenance. The CST complex regulates C-strand synthesis, defect in which may de-regulate telomerase activity or induce rapid telomere loss when telomerase is inhibited. (B) Shelterins protect telomere ends, and drugs that target shelterins may disrupt telomere protection. (C) T-oligos can induce senescence, apoptosis, and autophagy in vitro through a to-be-defined mechanism.

Telomere instability can arise from several mechanisms (Figure 1A). First, telomeres gradually shorten with each cell division as a part of normal cellular aging process due to the end-replication problem and telomere end resection [19]. When telomeres become critically short, short telomeres are sensed as damaged DNA, inducing cell cycle arrest that leads to senescence in normal somatic cells. If the p53 or Rb checkpoint pathway is deficient, cells continue to divide and the short unstable telomeres induce chromosome end-to-end fusions, leading to genome instability that drives oncogenesis. On the other hand, after a cell becomes a cancer cell, it needs a mechanism to maintain/restore telomere length in order to be immortal. To do so, cancer cells either express telomerase [20] or initiate a recombination-dependent alternative-lengthening-of-telomeres (ALT) pathway [21].

Pathological telomere shortening can arise due to problems with telomere synthesis such as defects in telomere replication, extension of G-strand by telomerase, and/or C-strand fill-in by DNA polymerase (Pol) [22–25]. In addition to telomere shortening, telomere instability can also occur when inappropriate secondary structures of telomere DNA, such as G-quadruplexes, form. Formation of these structures can interfere with telomere DNA synthesis by stalling replication forks at the telomeric region [26,27], leading to telomere fragility and possibly rapid loss of telomeres or elevated recombination [28–33].

In addition to telomere shortening, telomere instability can also result from telomere deprotection induced by deficiency in telomere binding proteins due to loss of DDR suppression and increased genomic rearrangements [34–36] (Figure 1B). For example, deletion of the shelterin TRF1 in mice activated DDR and increased sister-telomere fusions, chromosome end-to-end fusions, and telomere fragility [18]. Furthermore, telomere-induced chromosomal instability associated with TRF1 deletion contributed to early developing of skin tumorigenesis in a p53−/− background [18]. In addition, deletion of another shelterin, Pot1a, in mouse cells resulted in aberrant homologous recombination at telomeres and increased various cytogenetic abnormalities such as q-q arm chromosomal fusions without telomeric signals at fusion sites, isochromatid ring chromosomes completely devoid of telomeres, isochromatid ring chromosome without telomeres at sites of fusion, chromosomal fragment without telomeres, telomere fragments containing leading and lagging telomeric DNA, lagging telomeric fragments, and isochromatid breaks [36]. Importantly, the genomic instability resulting from Pot1a deletion was associated with increased tumorigenicity as Pot1a−/− MEF’s exhibited increased foci formation and skin tumor formation in a p53−/− background [36]. Furthermore, because DNA damage at telomeres is less likely to be repaired [37], accumulation of telomere damage can be a source of genomic instability in cells.

## Molecular Therapies to Directly Regulate Telomere Integrity

Because telomere stability contributes to replication immortality in cancer cells, targeting telomere stability by interfering with telomere synthesis or protection may yield new strategies for the treatment of cancer. Molecular targets that play a direct role in maintaining telomere integrity are telomere DNA, telomere synthesis, and telomere protection (Table 1).

### Telomere DNA Targets

#### G-quadruplex

Human telomere DNA is composed of a long DNA strand of guanine-rich tandem-repeat sequences (G-strand), and a shorter, complementary cytosine-rich strand (C-strand). Numerous proteins bind to telomere DNA and protect telomeres by preventing aberrant activation of DNA damage signaling at telomeres. However, certain small molecules can compete with these proteins for binding to telomere DNA and/or induce G-strand formation of the G-quadruplex (G4) secondary structure via Hoogsteen hydrogen bonding [38]. These small molecules, or G4 ligands, can disrupt telomere synthesis and maintenance by interfering with telomere elongation by telomerase [39] or by exposing telomere ends and activating DNA damage response. G4 ligands have shown promising anti-cancer activity *in vitro* and *in vivo*, leading to a search for small molecules that can selectively interact with and stabilize G-quadruplexes (for reviews, see [40,41]). Although several classes of G4 stabilizing molecules have been described [42], two severe limitations thus far with these compounds are lack of potency and relatively poor selectivity for binding to G-quadruplex versus duplex DNA [42]. Two newly described quadruplex-binding acridine ligands, BRACO-19 and RHPS4, however, can induce rapid replicative senescence in cancer cells and activate the same DNA damage response that follows DNA double-strand breaks [43]. One G4 stabilizing molecule, quarfloxin, was tested in phase II clinical trials for the treatment of neuroendocrine/carcinoid tumors, however, its mechanism of action seemed dependent, not on telomere instability, but on inhibition of G-quadruplexes involved in Pol I-dependent transcription [44].

### Telomere synthesis targets

#### Telomerase

Telomerase, a ribonucleoprotein DNA polymerase that synthesizes telomeres *de novo*, is over expressed in the majority of cancer cells [45]. Telomerase is composed of two core components: the reverse transcriptase hTERT and its associated template RNA, hTR. Currently, several classes of compounds that target either hTR or hTERT are being tested for their ability to inhibit telomerase and limit tumor growth. First, the most attractive emerging candidate to inhibit hTR, a N3’–P5’ thio-phosphoramidate named GRN163 or Imetelstat, competes with telomeric primer binding by hybridizing to hTR and inhibiting telomerase activity [46–49]. *In vitro* characterization of Imetelstat showed that it inhibits telomerase, induces telomere shortening, senescence, or apoptosis [50], and can reduce tumor growth in a DU145 mouse xenograft model of prostate cancer [50]. Excitingly, results from clinical trials show evidence for the potential utility of Imetelstat for treating hematological cancers and, possibly, a subset of patients whose solid lung tumors had short telomeres [51]. By contrast, other inhibitors target hTERT. A thymidine analogue, 3'-Azido-2',3'-dideoxythymidine (AZT) was found in CHO cells to incorporate preferentially at telomeres, and its incorporation can be telomerase-mediated [52]. Although the precise mechanism of action of AZT on telomerase remains elusive [52], it is interesting to note that treatment with AZT could produce effective inhibition of telomerase activity in varied cell lines as well as progressive telomere shortening and cytotoxic effects, although such effects are cell line dependent [53]. By contrast, another small molecule, BIBR1532, is a non-nucleosidic compound that directly binds and effectively inhibits hTERT by interfering with its enzymatic processivity [54], thus inducing telomere shortening and senescence in human cancer cells *in vitro* [55]. Compared to untreated controls, treatment with BIBR1532 of late-passage, telomere shortened, HT1080-derived xenografts in mice reduced initial tumor growth and decreased the incidence of tumors larger than 1000 mm3 [56]. The most recent work on BIBR1532 suggest that BIBR1532 may be particularly promising when given in combination with traditional chemotherapeutic agents [57,58].

#### CST complex

Composed of three proteins, Ctc1, Stn1, and Ten1, the human and mammalian CST complex binds to telomeres and is involved in telomere synthesis. In budding yeast, CST (Cdc13/Stn1/Ten1) acts as a telomere-capping complex that protects telomeres [59–61] and regulates telomere extension by telomerase [62]. Although in mammals, CST is not needed for telomere capping, the key function of mammalian CST is facilitating replication of telomere DNA, mediating C-strand fill-in [23,30,63,64], and inhibiting excessive telomerase extension of G-strand [65]. Interestingly, mammalian CST is also important for protecting the stability of non-telomeric DNA [23,30,63]. Such protection may be due to the role of CST in genomic DNA replication re-start after hydroxyurea (HU) induced fork stalling [63]. Although there are not any current cancer therapies that specifically target CST, the recent elucidation of a high-resolution structure of human Stn1-Ten1 complex may provide enough data for scientists to begin rational drug design of CST-targeting agents [66].

### Telomere protection targets

#### Shelterin proteins protect the ends of telomeres from genomic rearrangements

Another strategy to target telomere stability is to enhance or reactivate growth suppressive responses induced by telomere defects. Composed of the six proteins, TRF1, TRF2, TIN2, RAP1, POT1, and TPP1, the shelterin complex binds to telomeres, and protects telomeres by repressing DNA damage response at telomeres and preventing chromosome fusions [5,6,67]. Loss of shelterins de-represses DDR and allows non-homologous end joining (NHEJ) of chromosome ends, resulting in chromosome end-to-end fusions and genomic instability [17,18,35,36].

Targeting shelterin proteins as an approach of cancer therapies has emerged recently. For example, one shelterin Pot1 is targeted by a berberine derivative, Sysu-00692, which binds to Pot1 and interferes with the interaction between Pot1 and telomere DNA as observed by chromatin immunoprecipitation (ChIP) *in vitro* [68]. This compound also slightly inhibits telomerase in A549 cells and decreases cell proliferation in HL60 and A549 cancer cells [68].

Recent study indicates that another shelterin, TRF2, may be a potential target of cancer therapy. The potent anti-tumor drug gemcitabine is a nucleoside analogue that is currently approved for use against ovarian, breast, NSCLC, and pancreatic cancers. Although gemcitabine acts by incorporating into DNA in place of cytosine and inhibiting DNA replication, this drug can also induce XPF-dependent telomere loss by stabilizing TRF2 [69]. Interestingly, recent work shows that metronomic treatment with this drug has anti-angiogenic effects in a pancreatic cancer model [70]. However, caution is needed when applying drugs targeting shelterin components in anti-cancer therapy, because the shelterin proteins are essential for maintaining telomere stability in normal cells as well.

### T-loops may repress telomere-dysfunction induced foci (TIF) formation at telomere ends

The duplex lariat structure of the t-loop forms when the single strand 3’ G-overhang invades double-stranded telomeric repeats by base pairing with the C-strand and displacing the G-strand [4]. TRF2 has been shown *in vitro* to be sufficient for the formation of t-loop [3,71]. Recently, a three-state model of telomere protection was proposed [67]. Although hypothetical, according to this model, formation of the t-loop conceals linear chromosome ends into the telomeric repeats, thereby protecting chromosome ends from being recognized as damaged DNA. By contrast, loss of the t-loop would result in linearization of chromosome ends, activation of ATM and p53, and the formation of foci of aggregated DNA damage proteins. These foci, known as telomere-dysfunction induced foci or TIF, contain DNA repair factors such as 53BP1, -H2AX, Rad17, ATM, and the Mre11/Rad50/Nbs1 complex [72]. Thus, loss of the t-loop would result in linearization of chromosome ends, initiating DNA damage signaling and growth suppressive responses such as cell cycle arrest, senescence, or apoptosis in the absence of gross chromosomal rearrangements.

In support of this model, it has been shown that T-oligos, or oligonucleotides with homology to the G-rich strand of the t-loop, can induce senescence in human fibroblasts in a p53 and Rb dependent manner, consistent with telomere loop disruption [73]. Furthermore, treatment with T-oligos can induce apoptosis, senescence, or autophagy in a variety of cancer cell lines [74–79]. T-oligos can also inhibit angiogenesis in melanoma SCID xenografts [80]. Also, pre-treatment of cancer cells with T-oligos can confer radiosensitivity upon mammary carcinoma cells and in *in vivo* mouse mammary tumor models [81]. Importantly, the helicase WRN seems to play a role in T-oligo induced DNA damage response, as T-oligo treated fibroblasts deficient of WRN have reduced phosphorylation of p53 and H2AX [82]. Finally, the most recent work shows that inhibition of tankyrase can block DNA damage induction by T-oligos, suggesting that recruitment of shelterins away from telomeres by T-oligos may be needed for T-oligo induced DNA damage in MU, PM-WK, and MM-MC melanoma cells [83]. Although further work is needed to understand the mechanism of t-loops in telomere protection *in vivo*, these data suggest that T-oligos may induce growth suppression and DNA damage response by allowing an intermediate state of telomere deprotection.

## Molecular Therapies to Regulate Cell Fate in Response to Telomere Instability

### Telomere-binding proteins as information node between telomeres and cell fate decisions

At the interface of cell signaling and telomere regulation, telomere-binding proteins may integrate cell biological inputs to regulate telomere synthesis and protection, functioning as the “information node”. As outlined in an excellent recent review [84], telomere binding proteins are subject to post-translational modification (PTM) by kinases, sumoylases, acetylases, and ubiquitin ligases. Phosphorylation, sumoylation, acetylation, or ubiquitination can dynamically regulate telomere-binding protein function or activity in response to cellular stimuli. Thus, characterization of the PTM’s that regulate telomere binding protein function or activity may allow scientists to change the information at the telomere-cell node to alter cell fate.

Currently, a few PTM’s and their enzymes are known to regulate telomere function. First, shelterin components TRF1 and TRF2 are known to be phosphorylated, sumoylated, ubiquitinated, poly(ADP-ribosyl)ated (PARsylated), and methylated by a variety of enzymes [84,85]. These PTM’s of TRF1 and TRF2 regulate many aspects of TRF1 and TRF2 function at telomeres including telomeric DNA binding, TRF1 or TRF2 stability, protein-protein interactions, and priming of TRF1 for subsequent PTM's. Importantly, tankyrase, the poly(ADP-ribose) polymerase (PARP) that catalyzes TRF1 PARsylation and releases TRF1 from telomeres [86], is an emerging target for cancer therapy [87], and PARP inhibitors are in clinical trials for the treatment of cancer [88]. Excitingly, recent work also have shown that acetylation of TRF2 by p300 regulates TRF2 stability and telomere binding, and that overexpression of an acetyl-defective TRF2 mutant induces altered telomeres, telomeric DNA damage response, and senescence [89]. Another shelterin protein, TPP1, was recently shown to be phosphorylated by Cdk1, and this phosphorylation event appears to regulate telomerase recruitment to telomeres [90]. Because pharmacological inhibitors of many enzymes that catalyze PTM’s are commercially available, increased knowledge of the PTM's that regulate telomere synthesis and protection may allow scientists to alter the telomere status and replicative immortality of cancer cells by inhibiting the enzymes that catalyze the PTM's of telomere binding proteins.

## Outlook and Conclusions

### Telomeres as sensors of genotoxic stress

As telomere based therapies move from bench to bedside, understanding the targets and the mechanisms by which new therapies act will be important to optimizing their clinical use. Also, as the mechanisms that maintain telomere integrity are elucidated, learning how cells respond and adapt to changes in telomere status will yield a broader perspective on the role of telomeres in replicating cells. For example, it was recently shown that DNA damage at telomeric DNA is irreparable and causes persistent activation of DNA damage response [37]. Furthermore, in ageing and stress-induced senescence, telomeres can be preferred targets of DNA damage response [91]. Finally, work from the Karlseder lab recently showed that telomere deprotection was functionally distinct from genomic DNA damage response [92]. Together, these results suggest that, in contrast to genomic DNA, telomeres may function as specialized sensors of cellular [67] or genotoxic stress [7] that can induce the senescence or inhibit cell proliferation [7].

### Chemotherapy: beyond telomerase inhibition

A key mechanism driving replicative immortality in cancer cells is telomere elongation by telomerase [8], thus many current potential telomere based therapies have focused on targeting active components of telomerase. As knowledge of the regulation of telomere maintenance increases, however, other molecular targets that regulate telomere synthesis or cellular response to telomere status have emerged as potential regulators of replicative immortality in cancer cells. Importantly, these emerging regulators of telomere maintenance, telomeric DNA, the CST complex, the t-loop, or shelterins, may be potential targets for chemotherapeutic agents for cancer in the future.

## Figures and Tables

**Table 1:** Telomere integrity.

Target	Mechanism	Compound	Clinical Trial	Reference
**Telomere DNA**	inducing the formation of or stabilizing G-quadruplex	BRACO-19	No	43 (review)
		RHPS4	No	43 (review)
		Quarfloxin	Yes	44
**Telomerase**	hTR	Imetelstat	Yes	50, 66–69
	Inhibiting hTERT	AZT	approved for HIV	52, 53
		BIBR1532	No	54–58
**CST complex**	Ctc1, Stn1, Ten1	N/A	N/A	N/A
**Shelterins**	blocking Pot1 binding to telo- meres stabilizing TRF2	SYSU-00692	No	68
		Gemcitabine	approved for pancreatic cancer, breast can- cer, ovarian cancer, and lung cancer	69, 70
**T-loop**	destabilizing t-loop structure	t-oligos	No	73–83
**Post-translational Modifications**	TRF1-PARsylation	PARP inhibitor	Yes	88
